# Microbial Etiology of Pneumonia: Epidemiology, Diagnosis and Resistance Patterns

**DOI:** 10.3390/ijms17122120

**Published:** 2016-12-16

**Authors:** Catia Cilloniz, Ignacio Martin-Loeches, Carolina Garcia-Vidal, Alicia San Jose, Antoni Torres

**Affiliations:** 1 Department of Pneumology, Institut Clinic del Tórax, Hospital Clinic of Barcelona-Institut d’Investigacions Biomèdiques August Pi i Sunyer (IDIBAPS), University of Barcelona, Ciber de Enfermedades Respiratorias (CIBERES), 08036 Barcelona, Spain; catiacilloniz@yahoo.com (C.C.); sanjose@clinic.cat (A.S.J.); 2 Department of Clinical Medicine, Trinity Centre for Health Sciences, Multidisciplinary Intensive Care Research Organization (MICRO), Welcome Trust-HRB Clinical Research, St James’s Hospital, St James’s University Hospital, Dublin, Ireland; drmartinloeches@gmail.com; 3 Department of Infectious Diseases, Hospital Clinic of Barcelona, 08036 Barcelona, Spain; carolgv75@hotmail.com

**Keywords:** microbial etiology, pneumonia, diagnosis

## Abstract

Globally, pneumonia is a serious public health concern and a major cause of mortality and morbidity. Despite advances in antimicrobial therapies, microbiological diagnostic tests and prevention measures, pneumonia remains the main cause of death from infectious disease in the world. An important reason for the increased global mortality is the impact of pneumonia on chronic diseases, along with the increasing age of the population and the virulence factors of the causative microorganism. The increasing number of multidrug-resistant bacteria, difficult-to-treat microorganisms, and the emergence of new pathogens are a major problem for clinicians when deciding antimicrobial therapy. A key factor for managing and effectively guiding appropriate antimicrobial therapy is an understanding of the role of the different causative microorganisms in the etiology of pneumonia, since it has been shown that the adequacy of initial antimicrobial therapy is a key factor for prognosis in pneumonia. Furthermore, broad-spectrum antibiotic therapies are sometimes given until microbiological results are available and de-escalation cannot be performed quickly. This review provides an overview of microbial etiology, resistance patterns, epidemiology and microbial diagnosis of pneumonia.

## 1. Introduction

In 2014, the eighth cause of mortality in the United States reported by the National Center for Health Statistics was influenza and pneumonia together [1]. In children, pneumonia is the single largest infectious cause of death worldwide. In 2015, pneumonia killed 920,136 children under the age of 5, accounting for 15% of all deaths of children under five years old [2].

Pneumonia infection is the result of a complex process where the lower respiratory tract suffers the invasion of an infective microorganism. Pneumonia can be acquired in the community or acquired in the hospital environment, and can be transmitted by the aspiration of a pathogenic microorganism or by inhalation of a pathogenic microorganism. It is important to know the role of the pathogenic microorganism in the etiology of a pneumonia infection in order to provide adequate clinical and therapeutic management of the patient.

Globally, *Streptococcus pneumoniae* (pneumococcus) is the most common pathogen causing community-acquired pneumonia. Pneumococcus was considered one of the 9 bacteria of international concern in the recent worldwide report of global antibiotic resistance published by the World Health Organization (WHO) in 2014 [3]. On the other hand, a wide range of pathogens acquired either from the patient or from the hospital environment can cause nosocomial pneumonia. However, Gram-negative bacteria are more frequent than Gram-positive bacteria in these cases.

This review summarizes important features and management issues regarding the microbial etiology of pneumonia, focusing on epidemiology, pathogenesis, diagnostic testing and resistance patterns.

## 2. Microbial Etiology of Community-Acquired Pneumonia (CAP)

### 2.1. Epidemiology

In 2013, the Global Burden of Disease Study based on data from 188 countries around the world, reported that lower respiratory tract infection was the second most common cause of death [4]. In Europe, mortality rates for CAP vary widely from country to country, ranging from <1% to 48% [5].

The study by Jain et al. [6] about etiology of CAP showed an increased incidence of CAP with increasing patient age; the annual incidence of pneumonia in the USA was 24.8 cases per 10,000 adults, with the highest rates among adults aged between 65 and 79 years of age (63.0 cases per 10,000 adults) and those aged 80 years or older (164.3 cases per 10,000 adults).

The economic cost related to CAP remains high. A recently published Dutch study that included 195,372 CAP cases reported that the median costs of CAP cases were conditional on patient age and class of treatment care, varying from €344 per case for patients 0 to 9 years old treated as outpatients, to €10,284 per episode for 50–64 year olds admitted to the intensive care unit (ICU) [7].

### 2.2. Causative Microorganism

**Figure 1:**
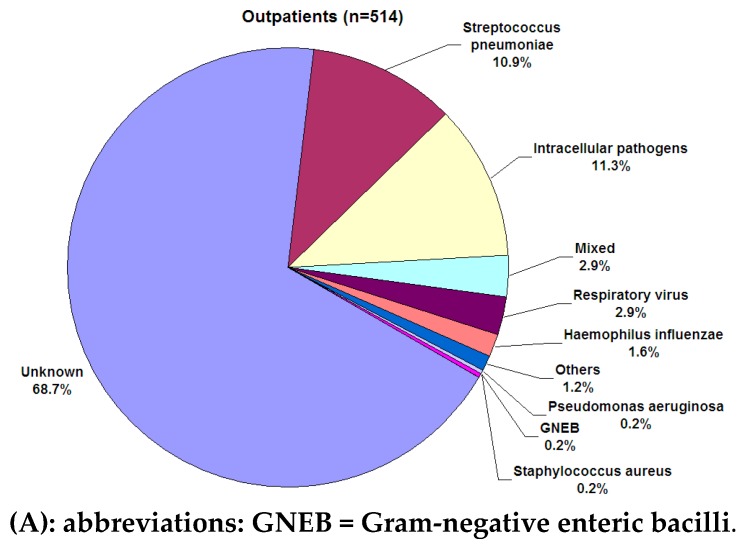

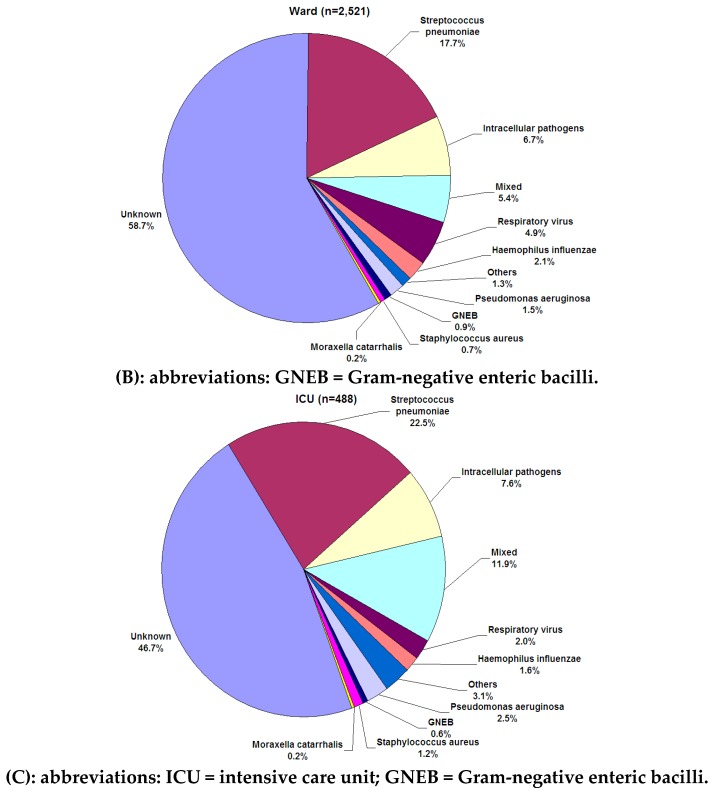
The most commonly identified pathogens among adults with Community-acquired pneumonia in Spain [8]. (**A**) Outpatients; (**B**) Patients Admitted to Ward; (**C**) Patients Admitted to Intensive Care Unit.

Several studies on the microbial etiology of CAP have been published in recent years [6,8]. Some of them showed that microbial causes of CAP differ according to the severity of disease at clinical presentation [9]. A Spanish study regarding the relationship of microbial etiology of CAP and severity, concluded that pneumococcus is the most frequent pathogen in all sites of care. The second most frequent group of pathogens was intracellular microorganisms, followed by polymicrobial cases (Figure 1).

Although microbiological diagnosis of CAP is fundamental to ensure appropriate antibiotic therapy, which is associated with decreasing mortality [10], microbial diagnosis of pneumonia is achieved in less than 50% of cases and antimicrobial therapy should be empirically administered in order to avoid the delay in establishing appropriate therapy, associated with significant mortality [11,12].

Globally, *Streptococcus pneumoniae* (pneumococcus) is widely accepted as being the most common pathogen in CAP, usually presented with acute symptoms of lower respiratory tract infection, historically called “typical presentation”. An estimated prevalence of 19.3% to 34% was reported for *S. pneumoniae* in Europe [13,14]. The diagnosis of pneumococcal pneumonia has increased significantly in recent years, mainly due to the introduction of the pneumococcal urine antigen test (routinely available from 2000 in most countries). Conversely, the incidence of pneumococcal pneumonia has probably decreased due to the introduction of pneumococcal vaccines, as well as the decreased rate of smoking in most countries [15].

Pneumococcus has several virulence factors; the most important being the polysaccharide capsule. Differences in chemical and antigenic composition of the pneumococcal capsule result in 93 different types or serotypes, approximately only 15 of which are involved in the majority of invasive infections. Serotype 3 is the most common serotype associated with adult pneumococcal infection and it has been associated with septic shock [16]. Serotypes such as 6A, 6B, 9V, 14, 19A, 19F and 23F are more common in children. However, in recent years, we have observed a change in the pneumococcal serotype and genotype distributions related to the introduction of pediatric pneumococcal conjugate vaccines (PCV7).

Intracellular pathogens are frequent causes of pneumonia [9,17], in these cases the clinical presentation is “atypical”, characterized by sub–acute symptoms, non-productive cough, low fever, normal white blood cells count and with frequency associated extrapulmonary manifestations. However, the incidence is variable, depending in part on the difficulties with microbiological cultures they grow poorly in standard culture media and culture requires expertise. Moreover, performing standard serologic tests on all patients with CAP is not common practice. The intracellular pathogens that are well-established as causes of CAP are: *Legionella pneumophila*, *Mycoplasma pneumoniae*, *Chlamydophila pneumoniae*, *Chlamydophila psittaci* and *Coxiella burnetii* [18,19]. No clinical features exist that make it possible to distinguish intracellular pathogens from classical pathogens (pneumococcus) in pneumonia, although extra-pulmonary manifestations are often associated with intracellular pathogens in CAP [20].

A recent review article [19] reported that severe CAP caused by intracellular pathogens accounts for approximately 1% to 7% of cases [6,8,21]. Since antimicrobial therapy for severe pneumonia is empiric and covers typical pathogens and the principal intracellular pathogens, results of microbiological diagnosis have an important relationship with the clinical prognosis of pneumonia.

Furthermore, co-infection with other pathogens is frequent in severe CAP cases. The study by Cilloniz et al. [22], which included 362 adult patients with severe CAP, found that 10% of the cases with defined microbial etiology were caused by intracellular pathogens. Co-infection involving intracellular pathogens and other pathogens was observed in 30% of cases caused by intracellular pathogens.

The biggest challenge for antimicrobial therapy for infection by intracellular pathogens is that most antibiotics are unable to access intracellular spaces and reach optimum therapeutic concentrations within the infected cells.

Respiratory viruses are considered the etiological agent in almost one-third of cases of CAP, in particular influenza viruses (A and B), rhinoviruses, parainfluenza viruses 1, 2 and 3, and coronaviruses. Globally, it is estimated that 100 million cases of viral pneumonia occur annually [23]. The improvement of molecular diagnostic techniques has demonstrated the increasing prevalence of viral pneumonia in recent years. The recently published study by Jain et al. [6] analyzing 2320 cases of pneumonia where an intensive microbiological diagnosis was applied (particularly viral molecular techniques), identified microbial etiology in 853 (38%) cases. The three main causal agents found were respiratory viruses (23%), bacterial etiology (11%) and co-infections (3%). One important issue regarding this study is that it showed that detection of respiratory viruses in CAP is much more frequent than was previously thought, thanks to molecular techniques.

Influenza virus (A/B) is usually self-limiting but severe complications (such as pneumonia) can occur, particularly in high-risk individuals (i.e., elderly patients with comorbidities or immunosuppressed patients). The influenza A (H1N1) pandemic of 2009–2010 gave us fresh knowledge of influenza. The World Health Organization estimated approximately 16,000 deaths between April 2009 and January 2010. The majority of these deaths corresponded to patients with underlying risk factors, such as metabolic dysfunctions, pregnancy, obesity and immunosuppression, contributing to worse outcomes [24,25].

In the past 20 years, respiratory syncytial virus (RSV) has been identified as an important cause of pneumonia in adults, especially in the elderly, where it has become the second most frequent viral cause. Overall, the rate of RSV as an etiology of CAP is between 2% and 5% throughout the year and between 5% and 14% during winter [26,27,28,29]. Adults with severe immunodeficiency are at particular risk of severe RSV infection [30,31].

Aspiration pneumonia is also a common cause of CAP. This etiology is frequently underestimated because of the difficulties in the diagnosis and in addition the microbiological tests are not usually applied. The most frequent microorganisms involved in aspiration are anaerobic bacteria and microaerophilic streptococci from the oral flora. Aspiration pneumonia may be the second most common etiology of CAP in the subset of patients over 80 years old [32].

### 2.3. Multidrug-Resistant (MDR) Pathogens in CAP

In CAP, in approximately 6% of the cases a MDR pathogen is involved, the most frequent described being *S. aureus* and *P. aeruginosa*. In a recent European study carry out by Aliberti et al. it was reported that MDR microorganisms were involved in 3.3% to 7.6% CAP cases in which the most commonly identified MDR pathogen was methicillin-resistant *S. aureus* (MRSA) [33]. Community-acquired methicillin-resistant *S. aureus* (CA-MRSA) has become an important CAP pathogen in endemic areas. The presence of the gene for the production of the toxin Panton-Valentine Leukocidin (PVL) is the main characteristic of CA-MRSA. PVL toxin causes leukocyte destruction and necrosis of the tissues. Presence of cavitation in the lung of a patient with pneumonia is a frequent characteristic in CA-MRSA, and is usually associated with skin lesions [34]. Since the recommendation of current international guidelines for severe CAP is empiric therapy with β-lactam with macrolide or fluoroquinolone, which may not provide adequate protection against MRSA, microbiological diagnosis of these cases is very important [35].

*P. aeruginosa* is not a frequent pathogen in CAP [8,36,37,38]. However, several studies have reported that in patients with severe CAP requiring ICU admission, *P. aeruginosa* was the causative agent in 1.8%–8.3% of the cases, with a case-fatality rate of between 50% and 100% [39,40,41,42,43]. A recently published study [44] found that 1% of cases were caused by MDR *P. aeruginosa*. The authors identified the use of prior antibiotic treatment as the only risk factor associated with CAP caused by MDR *P. aeruginosa*.

*S. pneumoniae*, the most common cause of CAP, has increased its resistance to several antibiotics (cephalosporins, macrolides and fluoroquinolones) worldwide in the last two decades [45,46,47]. Currently, between 20% and 30% of pneumococcus disease cases worldwide are MDR (resistant to more than three classes of antibiotics) [48,49]. Nevertheless, it is important to note that therapeutic failure involving β-lactams has not been reported among patients with pneumococcal pneumonia who are infected with pneumococci that are not susceptible to β-lactams. A reasonable explanation for this is that the mechanisms of resistance to penicillin are due to alterations in penicillin-binding proteins (PBPs), leading to decreased binding affinity. Pharmacodynamic studies show that a time above minimum inhibitory concentration (MIC) of about 40% of the dosing interval (T > 40% MIC) is predictive of bacteriological efficacy for β-lactams. Among the oral agents, aminopenicillins and cephalosporins are able to attain these levels in the lung for fully susceptible strains, and even for pneumococci with penicillin MICs of 2–4 g/mL.

A recent Spanish study [50] compared clinical outcomes in hospitalized patients with and without macrolide-resistant pneumococcus and reported that hospitalized patients for macrolide-resistant pneumococcal pneumonia have the same clinical presentation and outcomes as patients without macrolide-resistant pneumococcus.

## 3. Microbial Etiology of Hospital Acquired Pneumonia (HAP)

### 3.1. Epidemiology

Hospital-acquired (nosocomial) pneumonia (HAP) is defined as a pneumonia not incubating at the time of hospital admission and occurring 48 h or more after admission [51]. Ventilator associated pneumonia (VAP) is defined as a pneumonia occurring >48 h after endotracheal intubation [51].

HAP is the second most frequent nosocomial infection worldwide and is also considered the main cause of mortality for nosocomial infections. Regarding the consumption of antibiotics in the hospital, HAP accounts for approximately 50%, these data showing the impact on health resources [52,53,54,55]. Pneumonia that arises more than 48 to 72 h after endotracheal intubation is defined as Ventilator-associated pneumonia (VAP) and is considered the main nosocomial infection in the ICU [56,57,58]. VAP represented approximately 70% to 80% of all cases of HAP acquired in the ICU.

**Figure 2:**
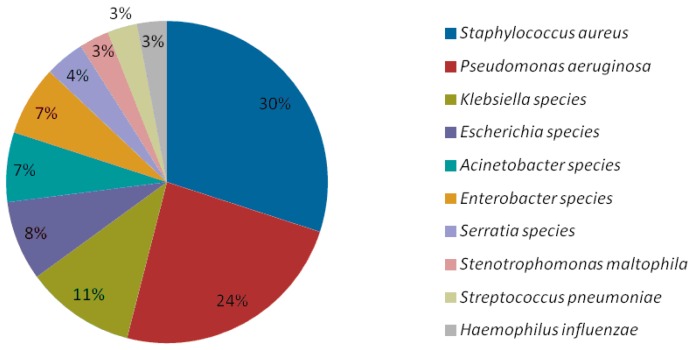
The most commonly identified pathogens in patients with Hospital-Acquired Pneumonia HABP/VABP (SENTRY Study).

HAP is divided into two groups according to the time of onset from admission [59] and this concept has been validated in several studies [60]. However, several subsequent studies have questioned the relationship between the timing of VAP and the risk of MDR pathogens [61,62]. In our opinion this concept is outdated. In addition, the recently published ATS/IDSA guidelines propose that the presence of risk factors for MDR should take precedence over the distribution between early and late onset pneumonia [51]. We used this concept in the present review only for a better comprehension (Figure 2).

(i) Early onset is defined as case development within the first four days of hospitalization. “Community” microorganisms are the main causes of these cases of pneumonia (methicillin-sensitive *Staphylococcus aureus*, *Streptococcus pneumoniae*, *Haemophilus influenzae*, and anaerobes. This kind of pneumonia is associated with better clinical prognosis.
(ii) Late onset is defined when pneumonia occurs after 5 days of hospitalization. The main pathogens involved in this kind of pneumonia are methicillin-resistant *S. aureus*, enteric gram negative bacilli, *P. aeruginosa* and non-fermenting bacteria (e.g., *A. baumannii*, *S. maltophilia*). Pneumonia caused by two or more pathogens (polymicrobial) is also frequent [59].
(iii) Early onset HAP tends to have a better prognosis than late onset HAP because of the association of the latter with MDR organisms.

### 3.2. Causative Microorganism

Most data concerning the etiology of HAP in ICU refer specially to the VAP population; data on etiology of non-ventilated intensive care acquired pneumonia (NV-ICUAP) remain limited. The study by Esperatti et al. [57] analyzed 315 episodes of ICU-acquired pneumonia and found that microbial etiology between VAP and NV-ICUAP were similar, with the only exception that they observed a higher proportion of *S. pneumoniae* in NV-ICUAP cases.

The recent article published by Koulenti et al. [63] on data from 27 ICUs in Europe from the EU-VAP/CAP study analyzed 2436 patients. Among NP cases, HAP occurred in 20.6%, VAP in 42.7% and very early-onset VAP (VE-VAP) in 12.7% of cases. Microbial diagnosis was possible in 69.5% of the suspected cases. The most frequent microorganisms reported were: *Enterobacteriaceae, S. aureus*, *P. aeruginosa* and *A. baumannii*, and a diagnosis of polymicrobial etiology was reported in 32% of cases. *Methicillin-susceptible S. aureus* (27.6% vs. 11.4%), *S. pneumoniae* (9.0% vs. 2.4%), *H. influenzae/M. catahrralis* (13.8% vs. 3.8%) were more frequent pathogens in early-onset pneumonia. The authors also reported a lower incidence of *A. baumannii* (11.0% vs. 26.5%) and a trend for a lower proportion of *P. aeruginosa* (17.9% vs. 26.1%, *p* = 0.09) in this group of cases. Other important data in this study showed that the dominant isolates differed between countries. They reported that in Spain, France, Belgium and Ireland, *S. aureus* was the dominant microorganism, whereas for Italy and Portugal it was *P. aeruginosa*, for Greece and Turkey it was *Acinetobacter* sp., and for Germany the dominant pathogen was *Escherichia coli.*

An important review article by Jones et al. on the results of the SENTRY Antimicrobial Surveillance Program (1997–2008) [64] was performed to establish which pathogens were most likely to cause Hospital acquired bacterial pneumonia (HABP) or ventilated acquired bacterial pneumonia (VABP). The study indicated that the 6 top pathogens causing 80% of HAP cases were: *S. aureus*, *P. aeruginosa*, *Klebsiella* spp., *Escherichia coli*, *Acinetobacter* spp., and *Enterobacter* spp. (Figure 2).

#### 3.2.1. Gram-Negative Pathogens

Gram-negative bacteria are implicated in 50% to 80% of the cases of HAP in an ICU [65]. The most frequent Gram-negative pathogens associated with HAP include:

(i) Pseudomonas aeruginosa.
(ii) Acinetobacter baumannii.
(iii) Haemophilus influenzae.
(iv) Enterobacteriaceae (*Klebsiella pneumoniae*, *E. coli*, Enterobacter species, Serratia species, Proteus species, etc.).

The study by Micek et al. [66] showed that mortality increased to 42% when the age of the patients increased, the Chalson score increased, there was inadequate initial antimicrobial treatment, and the only variable independent for predicted mortality was the use of vasopressors in the case of VAP where *P. aeruginosa* was isolated.

#### 3.2.2. Gram-Positive Pathogens

Gram-positive pathogens account for 20% to 30% of HAP cases [67]. *Methicillin-resistant* and *methicillin sensitive S. aureus*, pneumococcus and *Streptococcus* spp. are the most frequent pathogens.

#### 3.2.3. Polymicrobial Infection

Pneumonia caused by more than two pathogenic microorganism is defined as polymicrobial and approximately 30%–70% of VAP cases are considered to have polymicrobial origen [63,68,69]. The study by Combes et al. [69] found no differences regarding epidemiology data or clinical outcomes between monomicrobial cases or polymicrobial cases.

A study by Ferrer et al. [70], which included 441 cases, reported polymicrobial etiology of ICUAP in 16% of cases with confirmed microbiological etiology. The study also found that the presence of pleural effusion and the absence of chronic heart disease were associated with polymicrobial pneumonia. Polymicrobial etiology did not influence the outcome of ICUAP when empiric antibiotic treatment was frequently appropriate.

#### 3.2.4. Microbial Etiology of Early- and Late-Onset Pneumonia

**Figure 3:**
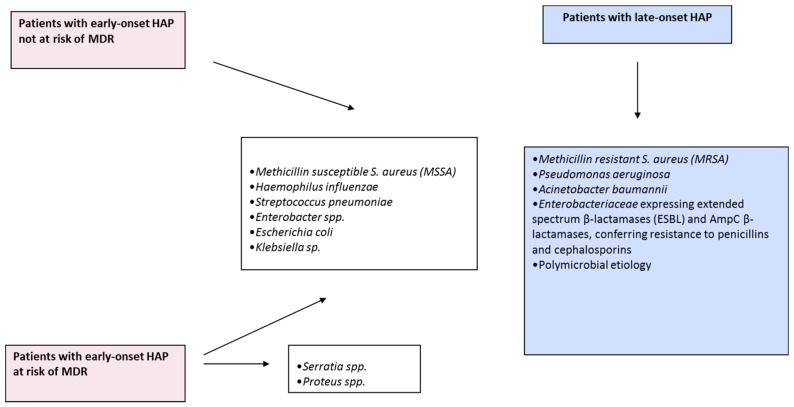
Pathogens associated with Early-Onset and Late-Onset Pneumonia. Abbreviations: MDR = multidrug-resistant pathogen; MRSA = methicillin resistant *S. aureus*, HAP = hospital acquired pneumonia; MSSA = methicillin sensitive *S. aureus*; ESBL = extended-spectrum β-lactamase.

HAP is divided into two groups according to the time of onset from admission [59] and this concept has been validated in several studies [60]. We used this concept in the present review only for a better reader comprehension. However, in our opinion this concept is outdated (Figure 3).

#### 3.2.5. Multidrug-Resistant Pathogens (MDR)

Antibiotic resistance is a global health problem with major consequences worldwide. The 2016 guidelines on HAP and VAP review several articles regarding risk factors for MDR pathogens. The guidelines summarize the following risk factors:

(i) Risk factors for MDR HAP: prior intravenous antibiotic treatment within 90 days;
(ii) Risk factors for MDR VAP: prior intravenous antibiotic treatment within 90 days; septic shock at time of VAP; ARDS preceding VAP; five or more days of hospitalization prior to the occurrence of VAP; acute renal replacement therapy prior to VAP onset.

The risk factors for specific pathogens were as follows:

Risk factors for *P. aeruginosa*; MRSA HAP/VAP: prior intravenous antibiotic treatment within 90 days.

The study by Martin-Loeches et al. [71] addressed the resistance patterns and outcomes in ICUAP in 343 patients. The authors reported that 35% of cases were caused by MDR pathogens. In this study, patients who developed ICUAP due to MDR pathogens showed higher ICU-mortality and remained in the ICU for a longer period compared with non-MDR cases.

## 4. Laboratory Diagnosis of Pneumonia

### 4.1. Clinical Samples to Be Collected

Since microbiological diagnosis of pneumonia is an important key factor for a better clinical outcome, it is very important to follow national and international guidelines. Recommendations regarding samples and diagnostic tests in pneumonia can be seen in Table 1.

#### 4.1.1. Community-Acquired Pneumonia

According to CAP guidelines, an optional microbiological diagnostic test in low to mild cases of CAP is recommended and in special situations it should be selected. In the case of severe CAP it is recommended to take blood cultures, sputum staining, sputum culture, and the urinary antigen test for *Legionella* and pneumococcus. There are some special situations where microbiological tests should be applied:

(i) Outpatients with failure of antibiotic therapy: sputum culture, urinary antigen test for *Legionella pneumophila* and *Streptococcus pneumoniae.*
(ii) Hospitalized patients with positive urinary antigen test for pneumococcus: sputum and blood culture.
(iii) Severe obstructive lung disease: sputum culture.
(iv) Pleural effusion: sputum and blood culture, urinary antigen test for pneumococcus and *Legionella*, pleural fluid culture.
(v) Cavitary infiltrates: sputum culture (bacteria, fungi and mycobacteria) and blood culture.
(vi) Active alcoholism: sputum and blood culture, urinary antigen test for *pneumococcus* and *Legionella.*
(vii) Severe CAP admitted to intensive care unit (ICU): sputum and blood culture, urinary antigen test for pneumococcus and *Legionella*, tracheal aspirate or bronchoalveolar lavage culture and viral studies also need to be performed.
(viii) Epidemiological factor or specific risk factors suggesting pathogen: urinary antigen test for *Legionella* (Legionnaires disease), influenza test during influenza season.

Microbiological diagnosis of CAP continues to be based on respiratory samples or blood culture. The main problems with these conventional methods are the low yield and long turnaround time (48–72 h) and the fact that previous antibiotic use affects microbiological results [72,73,74].

#### 4.1.2. Hospital Acquired Pneumonia

For cases of HAP (not-VAP), ATS/IDSA guidelines recommend that microbiological tests should be performed on respiratory samples obtained non-invasively (spontaneous expectoration, sputum induction, nasotracheal suctioning in a patient who is unable to cooperate to produce a sputum sample, and endotracheal aspiration in a patient with HAP who subsequently requires mechanical ventilation) [51].

For VAP cases, non-invasive sampling (endotracheal aspiration) with semi-quantitative cultures is recommended. Blood culture is also recommended for all patients with suspected VAP [51].

### 4.2. Diagnostic Testing for Pneumonia

#### 4.2.1. Conventional Microbiological Diagnosis

**Blood and pleural cultures:** Performing blood cultures in patients before a previous antimicrobial treatment has a high specificity but a low positivity (less than 20% of the cases) [35,75]. Pneumococcus is the main causative agent in blood cultures of patients with CAP [40].

The positivity of blood cultures in patients with HAP varies from 8% to 20%; the role of blood cultures in the diagnosis of VAP is limited because the spread of the infection to the blood occurs in <10% of cases [52].

In approximately 40% of CAP cases pleural effusion is present. Thoracentesis is recommended in these cases since empyema is considered a risk factor for poor outcome [76]. Pneumococcal antigen detection [77], or even molecular detection [78], are recommended in pleural fluid samples.

Falguera et al. [79] proposed the evaluation of six variables: liver disease, pleuritic pain, tachycardia, tachypnea, systolic hypotension and absence of prior antibiotic treatment, in order to predict bacteremia in CAP patients. In this score, for each predictive variable one point was assigned. A cut-off score of 2 in the derivation cohort was best for identification of the risk of bacteremia. On the other hand, rates of bacteremia were less than 8% for cases with score ≤1, whereas bacteremia presented in 14%–63% for cases with a score of 2 in the derivation cohort.

An important study on bacteremia caused by antibiotic-resistant pathogens (ARP) in CAP [80] reported that the risk factors for ARP bacteremia in CAP patients were; previous antibiotic use and C-reactive protein < 22.2 mg/dL. The authors also reported that inappropriate therapy was more frequent in ARP bacteremia compared with other bacteremias (27% and 3%, respectively, *p* < 0.001). The authors concluded that antibiotic therapy protected against bacteremia, but specifically increased the risk of bacteremia from ARP due to the inappropriate coverage of these pathogens.

**Sputum stain and culture:** Sputum sample collection is performed before patients initiate antimicrobial therapy. For an increase of microbiological diagnostic accuracy an adequate collection and transport of the sample is recommended; a good quality sample is considered when the sputum sample contains less than 10 epithelial cells and more than 25 lymphocyte cells.

In cases of pneumococcal CAP, the sensitivity of the Gram stain is approximately 80% [81] and the sensitivity of the Gram stain is 78% for pneumonia caused by *S. aureus*, with specificity between 93%–96% [82].

A presumptive diagnosis is considered when a pathogen is isolated from sputum culture since children <2 years old and patients with chronic pulmonary diseases frequently present oropharynx colonization by pneumococci. Endotracheal aspirate is the equivalent of sputum sample in VAP cases and both samples share the same criteria for quality. To distinguish colonization from infection a threshold ≥10^5^ colony forming units/mL is recommended in VAP cases [83].

**Urinary Antigen Detection:** Urinary Antigen Test: antigens from Legionella serotype 1 and pneumococcus are renally excreted and can be detected. This detection is not affected by the use of previous antimicrobial therapy. Sensitivity for pneumococcus detection ranges from 50% to 80% with reported specificity of 70% to 90%. The most common serogroup detected with urinary antigen test is *Legionella* serogroup 1, with sensitivity ranging from 70% to 90% and 99% of specificity.

Since there is a variance regarding the sensitivity and specificity of this test, it was proposed that concentration of urine may increment the sensitivity and specificity of this test [84]. A recent study by Saukkoriipi et al. [84] on the evaluation of the urinary antigen test in fresh, frozen and concentrated urine reported that, for fresh un-concentrated urine samples, the sensitivity for pneumococcal pneumonia was 63% and specificity was 97%. In the case of frozen and concentrated samples the sensitivity was 81% with 96% specificity.

#### 4.2.2. Molecular Microbiological Diagnosis

The development and implementation of molecular diagnostic tests for pneumonia has been a major advance in the microbiological diagnosis of respiratory pathogens in the last ten years [85,86,87,88]. Molecular tests help us identify a specific pathogen or help distinguish between bacterial and viral infection and provide information about antibiotic susceptibility patterns, monitor the response to antibiotic therapy, assess prognosis, aid antimicrobial stewardship, and give information for disease surveillance.

A recently published article [89] investigated the utility of a comprehensive molecular diagnostic approach encompassing 26 respiratory bacterial and viral pathogens, including bacterial quantification in patients with CAP. The study included 323 cases and reported that the use of molecular techniques in a single lower respiratory tract sample detected pathogen in 87% of pneumonia cases compared with 39% with culture-based methods. *H. influenzae* and *S. pneumoniae* were the most frequent pathogens reported in this study. Furthermore, 85% of the patients had received antimicrobials in the 72 h before admission; nonetheless PCR detected bacterial pathogens in 78% of these patients, whereas culture methods only detected 32% (*p* <0.001). The authors concluded that comprehensive molecular testing significantly improved pathogen detection in CAP, even in cases with previous antibiotic treatment.

The molecular platforms for pneumonia approved by the Food Drug Administration (FDA), and the most recent platforms are summarized in Table 2.

## 5. Conclusions

Microbial identification of pathogens causing pneumonia is an important issue for optimum clinical management of pneumonia and is a major challenge globally, given the expanding rate of multidrug-resistant pathogens and the emergence of new pathogens. However, despite the effort of collecting samples in pneumonia cases, approximately 50% of the cases remain without microbiological identification using conventional methods and recent studies have shown the importance of implementing new molecular platforms. We believe that conventional methods, together with molecular testing, will improve the microbiological diagnosis of pneumonia, thereby improving clinical management of cases, with shorter time to antibiotic therapy, better targeted antibiotic selection, more effective de-escalation and improved stewardship for pneumonia patients.

## Figures and Tables

**Table 1:** Samples and Diagnostic Testing in Pneumonia.

Condition of Pneumonia	Blood Cultures	Respiratory Samples	Urinary Antigen Test for Legionella/Pneumococcus	Comments
Outpatient		Sputum culture		Serology test when pathogens are suspected through epidemiological evidence
Hospitalized patients (ward)	×	×	×	Influenza test during influenza season
Hospitalized patients admitted to ICU	×	BAL/BAS in intubated patients	×	Serology test when pathogens are suspected through epidemiological evidence
Failure of outpatient antibiotic treatment		Sputum culture	×	Serology for intracellular pathogens
CAP cases who do not respond to treatment or suspicion of uncommon pathogens	×	BAL Mycobacterial and mycological culture Nasopharyngeal swab for respiratory viruses		
Hospital acquired pneumonia	×	×	×	Influenza test during influenza season
Ventilator associated pneumonia	×	BAS/BAL/mini BAL	×	

Abbreviations: BAL (bronchoalveolar lavage); BAS (bronchoaspirate); ICU (intensive care unit); CAP (community-acquired pneumonia) [3,53].

**Table 2:** Molecular platforms for pneumonia [79,80,81,82,83].

Platform	Pathogens	Technology	Sensibility/Specificity	Time	Sample	Advantages	Disadvantages	Approved
Curetis Unyvero P50 Pneumonia	18 bacterial and fungal pathogens 22 antibiotic resistance markers	Multiplex-PCR cartridge system	81%/99%	4 h	Sputum, BAL, BAS	Detection of resistant patterns	Test limited to a two samples test per run. A relatively large amount of hands-on time	Under FDA/EC/Under Singapore Registration/Under Chinese Registration
GeneXpertMRSA/SA	Methicillin-resistant *S. aureus* (MRSA) methicillin-sensitive *S. aureus* (MSSA)	Multiplex-PCR	99%/72%	1 h	Blood, Nasal swabs	Minimal technical expertise	Only detects MRSA/SA	FDA/EC
MALDI-TOF	200 microorganisms	Mass spectrometry, identification of microorganisms directly from colonies of bacteria and fungi	99%–100%/97%–100%	0.5–1 min	Colonies, positive blood cultures, direct samples such as urine	Rapid and accurate approach to detect microorganism	Lack of standardized assay conditions	
GeneXpert Flu Assay	Influenza A/B (A/2009H1)	multiplex-PCR	97%–100%/100%	1 h	Nasopharyngeal swabs, nasal aspirates and washes	Minimal technical expertise	Only detects influenza viruses	FDA/EC
GeneXpert Flu/RSV Assay	Influenza A/B/RSV	Multiplex-PCR	97%–100%/100%	0.5–1 h	Nasopharyngeal swabs, nasal aspirates and washes	Minimal technical expertise	Only detects influenza viruses and RSV	FDA/EC
FilmArray Respiratory Panel	Adenovirus; coronaviruses 229E, OC43, NL63, HKU1; metapneumovirus; influenza A, H3, H1, 2009 H1; parainfluenza viruses 1, 2, 3, 4; RSV; rhinovirus/enterovirus *B. pertussis*, *M. pneumoniae*, *C. pneumoniae*	An unprocessed biologic/clinical sample is subjected to nucleic acid purification, reverse transcription, a high-order nested multiplex PCR and DNA melting curve analysis	84%–100%/98%–100%	1 h	Nasopharyngeal Swab	Minimal technical expertise required	Test limited to a single patient test per run. Decreased sensitivity for some adenovirus types	FDA/ EC

Abbreviations: PCR = polymerase chain reaction; MSSA = methicillin sensitive *S. aureus*; MRSA = methicillin resistant *S. aureus*; RSV = respiratory syncitial virus; FDA = Food Drug Administrarion; EC = European Community; DNA = Deoxyribonucleic acid; MALDI-TOF = matrix-assisted laser desorption/ionization time-of-flight.

## Abbreviation

CAP	Community-acquired pneumonia
EPIC	Etiology of Pneumonia in the Community
ARP	Antibiotic resistant pathogen
ICU	Intensive care unit
RSV	Respiratory syncitial virus
MDR	Multidrug-resistant
MRSA	Methicillin-resistant *S. aureus*
MSSA	Methicillin-sensitive *S. aureus*
CA-MRSA	Community-acquired methicillin-resistant *S. aureus*
PVL	Panton-Valentine leukocidin
PBPs	Penicillin-binding proteins
MIC	Minimum inhibitory concentration
HAP	Hospital Acquired Pneumonia
VAP	Ventilator associated pneumonia
NV-ICUAP	Non-ventilated intensive care acquired pneumonia
VE-VAP	Very early-onset VAP
HABP	Hospital acquired bacterial pneumonia
VABP	Ventilated acquired bacterial pneumonia
ESBL	Extended spectrum β-lactamases
CFU	Colony-forming units
